# Serum nicotinamide phosphoribosyltransferase as a novel biomarker for non-traumatic osteonecrosis of the femoral head

**DOI:** 10.1186/s13018-022-03417-6

**Published:** 2022-11-28

**Authors:** Shiying Wang, Huixian Zhan, Liping Xu, Baoxiang Zhao

**Affiliations:** 1 Department of Orthopedics, Linyi People’s Hospital, Linyi, 276000 Shandong China; 2 Guangzhou University of Chinese Medicine, Guangzhou, 513000 Guangdong China; 3 Department of Laboratory Medicine, Qingdao Central Hospital, Second Affiliated Hospital of Qingdao University, Qingdao, 266042 Shandong China

**Keywords:** Non-traumatic osteonecrosis of the femoral head, Disease severity, Nicotinamide phosphoribosyltransferase, Biomarker

## Abstract

**Objective:**

The aim of this study was to investigate the potential role of serum nicotinamide phosphoribosyltransferase (NAMPT) in non-traumatic osteonecrosis of femoral head (NONFH).

**Methods:**

A total of 113 NONFH patients and 81 healthy individuals were included in this study. The NAMPT levels in serum were measured by a commercial enzyme-linked immunosorbent assay kit. Radiographic progression was determined using Association Research Circulation Osseous (ARCO) classification system. Clinical severity was assessed by Harris hip score (HHS) and visual analogue scale (VAS). Correlations between serum NAMPT and radiographic progression as well as clinical severity were evaluated statistically. Receiver operating characteristic (ROC) curves were performed to evaluate the diagnostic values of NAMPT in NONFH potential and disease severity.

**Results:**

The serum NAMPT levels in NONFH patients were significantly lower than that in healthy controls. There were no significant differences among alcohol-induced group, steroids-induced group, and idiopathic group. NONFH patients with ARCO stage 4 had significant lower serum NAMPT levels in comparisons with ARCO stage 3 and 2, respectively. Lower serum NAMPT levels were also observed in bilateral NONFH cases compared with cases with unilateral NONFH. In addition, serum NAMPT was negatively correlated with ARCO stages and VAS scores, and positively correlated with HHS. ROC curve analysis indicated that serum NAMPT may serve as a novel biomarker for diagnosing early NONFH and for monitoring disease severity.

**Conclusions:**

Our results suggest that serum NAMPT may serve as a novel biomarker for NONFH potential and disease severity.

## Introduction

Non-traumatic osteonecrosis of the femoral head (NONFH) is a degenerative condition characterized by bone cell necrosis and trabecular bone fracture, which usually progress to the eventual collapse of the femoral head [1]. More than 20 million people have been diagnosed as NONFH worldwide, with the increase of 15,000 to 20,000 new cases annually in the USA, and 100,000 to 200,000 in China [2, 3]. NONFH predominantly affects young patients in their third to fifth decades of life [4]. Preservation procedures of the femoral head are the first choice in young patients without collapse [5]. Total hip arthroplasty (THA) remains the main treatment for NONFH patients in late stages. Approximately 65%–70% of patients with advanced NONFH require THA [6, 7]. The possibility of frequent revisions could be challenging [8, 9]. Meanwhile, early diagnosis of NONFH is yet challenging, because it can frequently be asymptomatic in early stages [10]. A large number of studies suggested that the development of NONFH is associated with microcirculation impairment, decreased osteogenic differentiation, and increased adipogenesis differentiation [11, 12]. Once the femoral head collapses, it is difficult to reverse. In addition, it is difficult to screen for NONFH in the early phase using routine imaging approaches [13, 14]. Therefore, there is an urgent need to find novel biomarkers for diagnosing early NONFH and monitoring disease severity.

Nicotinamide phosphoribosyltransferase (NAMPT), also known as pre-B cell colony-enhancing factor or visfatin, is considered as a pleiotropic protein with many roles in physiology and pathology. NAMPT was shown to play key roles in modulating inflammation, apoptosis, insulin resistance, and oxidative stress response in different metabolic disorders and cancers [15–17]. In recent years, the role of NAMPT in bone metabolism has been partially investigated. In vitro studies demonstrated that NAMPT can promote osteogenic differentiation in preosteoblasts and bone marrow mesenchymal stem cells (BMSCs) [18, 19]. In addition, NAMPT can be detected in extracellular environments and in the human circulation. Abnormal circulating levels of NAMPT were associated with various musculoskeletal disorders such as osteoporosis, osteoarthritis, and rheumatoid arthritis, suggesting an emerging role of NAMPT as an important biomarker and therapeutic target [20–22].

The studies mentioned above suggest that NAMPT may play a pivotal role in the development of NONFH. Nevertheless, no previous study has investigated the correlation between serum NAMPT levels and NONFH. In this study, we examined NAMPT levels in serum of NONFH subjects and healthy controls to investigate its utility as a biomarker for early diagnosis and disease severity in NONFH.

## Study subjects and methods

### Study subjects

The study was approved by the Ethics Committee of Linyi People’s Hospital (No. YX200342) and was conducted in accordance with the ethical standards of the Helsinki Declaration. A retrospective analysis was conducted on a prospectively-maintained institutional database. A total of 113 consecutive NONFH patients were included between July 2020 and May 2021. The diagnosis of NONFH was based on the history, physical examination, and imaging findings. The inclusion criteria for case subjects were as follows: (1) age ≥ 18 years; (2) diagnosed as NONFH according to a Chinese Guideline for clinical diagnosis and treatment of NONFH in adults (2019 version) [23]. Exclusion criteria were as follows: (1) history of hip, or femur surgery or trauma, (2) history of malignancy, cardiovascular diseases, and metabolic bone disease such as rheumatoid arthritis, (3) involved in other clinical trials within 3 months, (4) treatment with medication affecting bone metabolism. The following demographic and clinical data was derived from the database which was collected at admission: gender, age, height, weight, and history of conventional risk factors (smoking, alcohol abuse, and steroids use).

### Propensity score matching

Propensity score matching (PSM) is a commonly used statistical method to reduce selection bias and balance the baseline characteristics in retrospective study where the benefit of randomization is not possible [24–26]. PSM analysis was performed in this study with the MatchIt package with the coarsened exact matching method in R software (version 4.2.1 for Windows). The covariates included age, gender, and body mass index (BMI). As a result, a total of 81 age-, gender- and BMI-matched healthy controls were successfully matched at the medical examination center of Linyi People’s Hospital with no history of osteonecrosis and none of the exclusion criteria described above.

### Laboratory assessment

Blood samples from all participants were collected after an overnight fast in plain in tubes with a coagulating agent. Samples were then centrifuged to obtain serum, which was separated and kept at − 80 °C prior to examination. NAMPT concentrations in serum were detected by a commercial enzyme-linked immunosorbent assay (ELISA) kit (Cat. No. CSB-E08940h, Cusabio, Wuhan, China) according to manufacturer’s instructions. The detection range was from 0.625 to 40 ng/mL. The intra- and inter-assay coefficients of variation were < 8% and < 10%, respectively.

### Radiographic assessment

The severity of radiographic progression of NONFH was evaluated using the Association Research Circulation Osseous (ARCO) 4-staged system [27]: stage 1, X-ray is normal, but either magnetic resonance imaging (MRI) or bone scan is positive; stage 2, X-ray is abnormal (subtle signs of osteosclerosis, focal osteoporosis, or cystic change in the femoral head) but without any evidence of subchondral fracture, fracture in the necrotic portion, or flattening of the femoral head; stage 3, fracture in the subchondral or necrotic zone as seen on X-ray, and stage 4, X-ray evidence of osteoarthritis with accompanying joint space narrowing, acetabular changes, and/or joint destruction.

### Definition of clinical severity

The visual analogue scale (VAS) ranged from 0 to 10 was used for evaluating degree of pain in NONFH patients, where 0 represents no pain and 10 indicates extremely pain. The VAS scores are broadly used for severity of pain in various diseases including NONFH. The Harris Hip Score (HHS) was used for measuring the symptomatic severity. The HHS comprises a total score of 100 points, the higher the score, the more satisfactory the hip function [28].

### Statistical analysis

Statistical analysis was performed using SPSS Statistics software version 21.0 and Graphpad Prism 6.0. Mean ± standard deviation was used to describe continuous variables, whereas frequencies were used to describe categorical variables. Statistical normality was assessed using the Kolmogorov–Smirnov test. The statistical significance of differences in continuous variables was assessed using the independent-sample *t* test for normally distributed data or the Mann–Whitney *U* test for skewed data. The chi-square test was used to compare categorical variables. Pearson or Spearman correlation was used for the correlation analysis between NAMPT and radiographic progression as well as clinical severity, where appropriate. ROC curves analysis for serum NAMPT was employed to determine the diagnostic values for NONFH and disease severity. *P* values less than 0.05 were considered as statistically significant.

## Results

### Basic characteristics of participants

Demographic characteristics of patients and controls are listed in Table 1. A total of 113 NONFH patients and 81 healthy controls were included in this study. The NONFH group consists of 86 men and 27 women, while the healthy control group consists of 59 men and 22 women. The average age was (51.67 ± 12.55) years in NONFH patients and (51.16 ± 10.70) years in healthy controls, respectively. The average BMI was (24.39 ± 2.73) kg/m^2^ in NONFH group and (24.76 ± 3.36) kg/m^2^ in healthy controls, respectively. No significant differences were observed in gender, age and BMI between two groups.

**Table 1:** Demographic data for NONFH patients and healthy controls

	NONFH patients (*n* = 113)	Healthy controls (*n* = 81)	*P* value
Gender (male/female)	86/27	59/22	0.606
Age (year)	51.67 ± 12.55	51.16 ± 10.70	0.764
BMI (kg/m^2^)	24.39 ± 2.73	24.76 ± 3.36	0.431
VAS	3.99 ± 1.98		
HHS	60.92 ± 19.50		
ARCO stage (I/II/III/IV)	4/36/36/37		
Etiology (steroid/alcohol/idiopathic)	40/22/51		
Side (unilateral/bilateral)	44/59		

### Comparisons of serum NAMPT levels

**Fig. 1:**
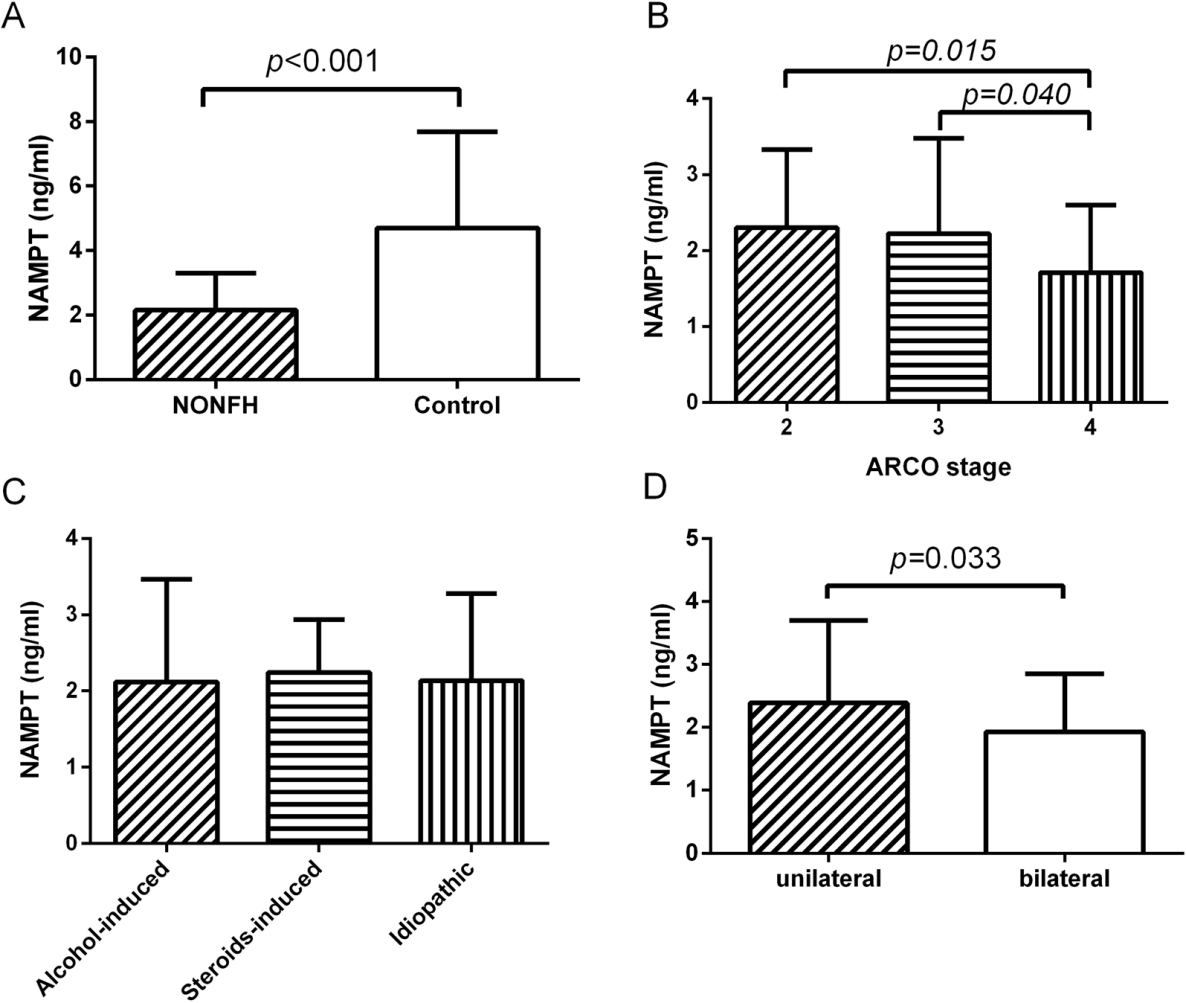
**A** Comparison of serum NAMPT levels between NONFH patients and healthy controls. **B** Comparisons of serum NAMPT levels among different ARCO stages. **C** Comparisons of serum NAMPT levels among alcohol induced, steroid-induced and idiopathic NONFH. **D** Comparison of serum NAMPT levels between unilateral and bilateral NONFH

As demonstrated in Fig. 1, serum NAMPT levels were significantly lower in NONFH patients (2.15 ± 1.14 ng/mL) than that in healthy controls (4.69 ± 2.99 ng/mL) (Fig. 1A). Also, serum NAMPT levels were markedly lower in ARCO stage 4 (1.71 ± 0.89 ng/mL) compared with those in ARCO stage 3 (2.22 ± 1.25 ng/mL) and ARCO stage 2 (2.30 ± 1.03 ng/mL) (Fig. 1B). No significant differences were observed in serum NAMPT levels among patients with different etiologies (alcohol-induced 2.12 ± 1.34 ng/mL vs steroids-induced group 2.24 ± 0.69 ng/mL group vs idiopathic group 2.14 ± 1.14 ng/mL) (Fig. 1C). Unexpectedly, lower serum NAMPT levels were observed in bilateral NONFH cases (1.93 ± 0.92 ng/mL) in comparison with cases with unilateral NONFH (2.39 ± 1.31 ng/mL) (*P* = 0.033) (Fig. 1D).

### Correlation analysis of serum NAMPT with disease severity and other indices

**Fig. 2:**
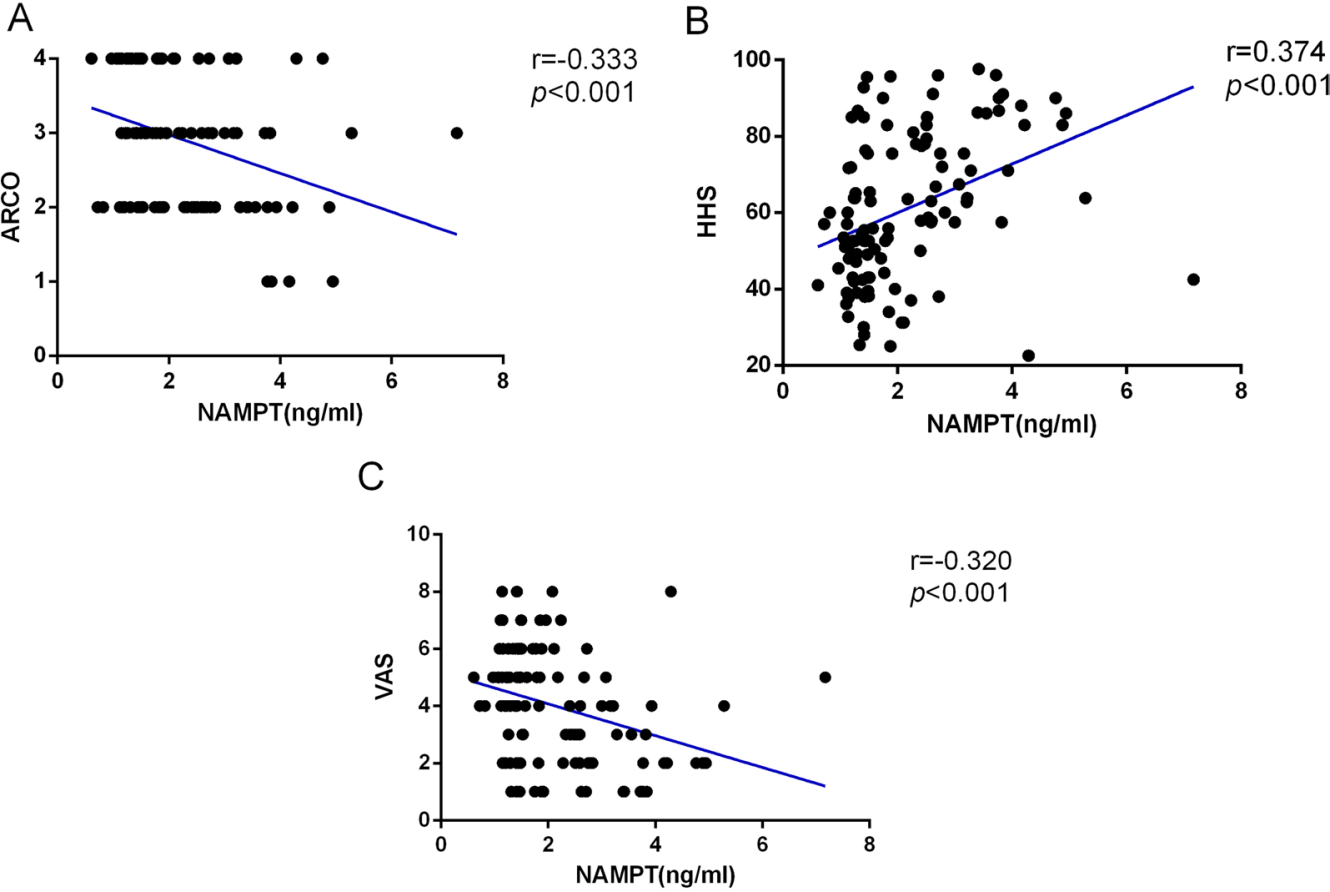
**A** Correlation of serum NAMPT levels with ARCO stages. **B** Correlation of serum NAMPT levels with HHS. **C** Correlation of serum NAMPT levels with VAS

We investigated the correlation of serum NAMPT with disease severity and other indices including HHS and VAS to illustrate whether serum NAMPT is related to clinical manifestations. As shown in Fig. 2, the serum NAMPT levels were negatively correlated with ARCO stages (*r* = − 0.333, *P* < 0.001) (Fig. 2A) and VAS scores (*r* = − 0.320, *P* < 0.001) (Fig. 2C), respectively. The serum NAMPT levels also positively correlated with HHS scores (*r* = 0.374, *P* = 0.033) (Fig. 2B).

### Diagnostic performance of serum NAMPT

**Fig. 3:**
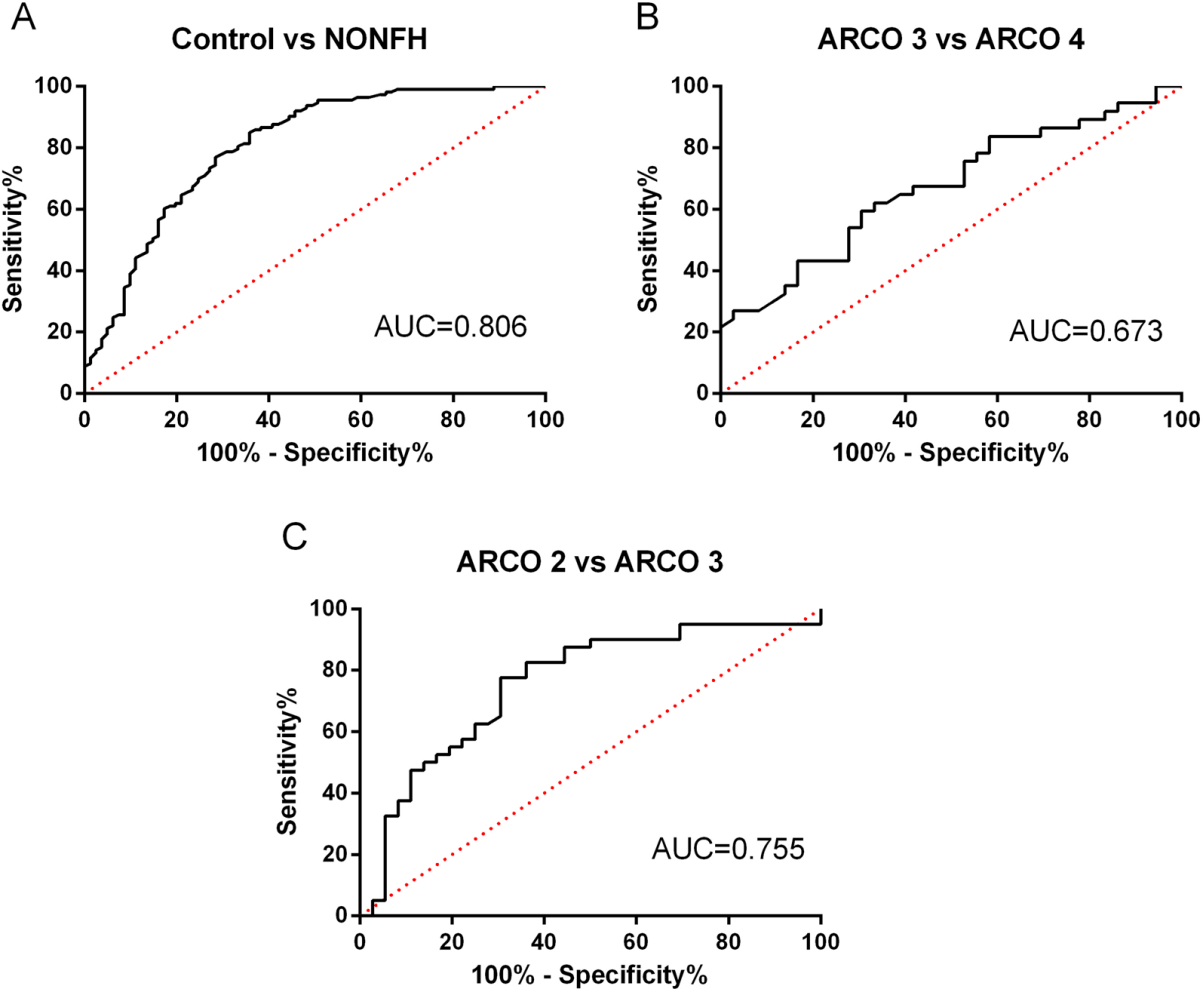
**A** ROC curve analysis of serum NAMPT levels with regard to healthy controls vs NONFH cases. **B** ROC curve analysis of serum NAMPT levels with regard to ARCO 3 vs ARCO 4. **C** ROC curve analysis of serum NAMPT levels with regard to ARCO 2 vs ARCO 3

We further performed ROC curve analysis to explore the diagnostic values of serum NAMPT for NONFH potential and disease severity. As shown in Fig. 3, the results reveal ROC of serum NAMPT between NONFH patients and healthy controls with an area under curve (AUC) of 0.806. At cutoff of 2.735 ng/mL, the sensitivity was 76.99%, the specificity was 71.60%, and the accuracy was 74.74%. The AUC was 0.673 for ROC of serum NAMPT with regard to ARCO stage 3 vs stage 4 and 0.755 for ROC of serum NAMPT with regard to ARCO stage 2 vs stage 3, respectively. These findings indicate that serum NAMPT may serve as a potential biomarker for early diagnosis and disease severity prediction of NONFH.

## Discussion

To the best of our knowledge, the present study for the first time explored the correlation between serum NAMPT levels and NONFH patients to test the diagnostic value of serum NAMPT for NONFH potential and disease severity. Notably, we found that serum NAMPT levels were significantly lower in NONFH patients compared to healthy controls. This suggested that NAMPT are involved in the pathogenesis of NONFH.

NAMPT is a 52 kDa protein broadly expressed in the musculoskeletal system, including muscle, bone, synovium, and cartilage [29]. The gene for NAMPT is located on the long arm of chromosome 7 (7q22.2) [30]. NAMPT is proposed as an important pro-inflammatory mediator. In recent years, the concentrations of circulating NAMPT have been widely studied in many types of cancers, metabolic conditions and chronic inflammatory diseases. NAMPT is considered to be a biomarker in various disorders as well. In breast cancer patients, the levels of serum NAMPT were higher compared with healthy individuals, suggesting that serum NAMPT may be a diagnostic parameter for breast cancer [31]. Compared to normal control subjects, higher concentration of circulating NAMPT was also observed in rheumatoid arthritis [32], osteoarthritis [33], and osteoporosis [22]. BMSCs from patients with NONFH exhibited weaker ability of osteogenic differentiation [34]. It is noteworthy that NAMPT may serve as the marker for osteogenic differentiation of BMSCs [18]. We speculate that the weaker osteogenic differentiation ability is a potential cause of the decreased NAMPT levels in NONFH patients. Further experimental studies are still needed to confirm the speculation.

In this study, we observed no differences of serum NAMPT levels among alcohol-induced group, steroids-induced group, and idiopathic group, implicating the change of serum NAMPT levels in NONFH regardless of etiology. Besides, decreased serum NAMPT levels were observed in ARCO stage 4 compared with those in ARCO stage 3 and 2. Interestingly, lower serum NAMPT levels were also detected in bilateral NONFH cases compared to unilateral NONFH ones. Furthermore, our results revealed a negative correlation of serum NAMPT with ARCO stages and VAS scores as well as a positive correlation with HHS scores. A better understanding of the molecular mechanism of the above correlation may be achieved by investigating the potential role of NAMPT in bone anabolism. Briana et al. [35] reported that fetal circulating levels of NAMPT were positively correlated with bone anabolic markers. Moreover, NAMPT knock-down or inhibition in mouse BMSCs reduced the osteoblastogenesis, alkaline phosphatase activity, matrix mineralization, and the expression of osteoblast differentiation markers.

Finally, ROC curve analysis of serum NAMPT to detect NONFH revealed a relative high diagnostic efficacy with an AUC 0.806. The AUC was 0.673 for ROC of serum NAMPT with regard to ARCO stage 3 vs stage 4 and 0.755 for ROC of serum NAMPT with regard to ARCO stage 2 vs stage 3, respectively. Our results regarding AUC are better than those in several NONFH studies. Liu et al. [36] reported an AUC of 0.645 for ROC of serum vasoactive intestinal peptide (VIP) with regard to ARCO stage 3 vs stage 4 and 0.721 for ROC of serum VIP with regard to ARCO stage 2 vs stage 3, respectively. Jiang et al. [37] reported the AUC was 0.695 for ROC of plasma Circ CDR1as with regard to ARCO stage 3 vs stage 4 and 0.635 for ROC of plasma Circ CDR1as with regard to ARCO stage 1/2 vs stage 3, respectively. Taken together, our results indicated that serum NAMPT may serve as an effective biomarker for diagnosing early NONFH and for monitoring disease severity.

There are still several limitations that should be taken into account when interpreting the results. First, this is a cross-sectional study, so the causal relationship between serum levels of NAMPT and development of ONFH could not be illuminated. Therefore, further experimental studies are still needed to explore the detailed mechanisms in NONFH. Second, the study was performed on Chinese (Han) populations and cannot be generalized to all populations.

## Conclusion

The serum NAMPT levels were significantly lower in NONFH patients compared to healthy individuals. Serum NAMPT may serve as a novel biomarker for NONFH potential and disease severity.

## Data Availability

The datasets used and/or analyzed during the current study are available from the corresponding author on reasonable request.
